# Brief Report: Can Fecal Microbiota Transplantation Treat Depression?

**DOI:** 10.1002/jgh3.70336

**Published:** 2026-01-22

**Authors:** Sara Benterkia, Jenny Blythe

**Affiliations:** ^1^ Barts and The London Medical School Queen Mary University of London London UK

**Keywords:** depression, fecal microbiota transplantation, FMT, TRD, treatment resistant depression

## 1 Introduction

Major depressive disorder (MDD) is a debilitating condition affecting around 280 million people worldwide [1]. Despite advances in pharmacological and psychotherapeutic treatments, around 30% of patients develop treatment‐resistant depression, characterized by marked functional impairment, diminished quality of life, and increased suicidality. The global prevalence of depression continues to rise, with a further 25% increase reported since the Covid‐19 pandemic [2]. This growing burden underscores the need for novel therapeutic approaches, including investigation of the gut–brain axis (GBA)—the bidirectional communication between the central nervous system and gastrointestinal microbiota. This report reviews pre‐clinical and clinical evidence evaluating fecal microbiota transplantation (FMT) as a potential treatment for depression.

## 2 Background

Disruption of the gut microbiota, termed dysbiosis, has been implicated in the development of psychiatric conditions including depression [1]. Dysbiosis contributes to depression through reduced production of short‐chain fatty acids (SCFAs), which normally promote anti‐inflammatory cytokines and support neurological functions such as serotonin synthesis [1]. Inflammatory microbes drive gut inflammation and increase intestinal permeability via cytokine release (e.g., TNF‐α, IFN‐γ, IL‐6) [1]. SCFA supplementation in mice has demonstrated antidepressant effects, supporting a link between dysbiosis‐driven inflammation and depression [1].

FMT, already approved for refractory *Clostridium difficile* infection, has been proposed as a novel method to restore microbial balance and improve mental health outcomes [3]. It aims to restore a healthy microbial ecosystem by transferring stool from a screened, healthy donor into the gastrointestinal tract of a recipient. The donor selection process includes rigorous screening, followed by administration via colonoscopy, enema, or encapsulated oral formulations. In the context of depression, FMT is hypothesized to reverse dysbiosis, reduce inflammation, and enhance neurotransmitter function [3].

**FIGURE 1:**
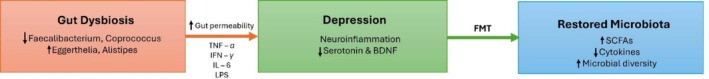
GBA pathway summary (diagram author's own).

Figure 1 summarizes these GBA pathways [1, 4, 5, 6].

## 3 Methods: Review of the Literature

A search of the PubMed database was undertaken to identify studies examining the relationship between FMT and depression. Search terms used were (“faecal microbiota transplantation” OR “fecal/faecal microbiota transplantation” OR “FMT”) AND (“depression” OR “treatment‐resistant depression” OR “TRD” OR “mental health” OR “gut–brain axis” OR “gut dysbiosis” OR “microbiome”), including all types of studies published in the English language since 2016 (the year reflecting the novel use of considering FMT for depressive conditions). The search was keyword‐based but cross‐checked with MeSH headings (e.g., depressive disorder, fecal microbe transplantation) for consistency. The search found one pre‐clinical review, two randomized controlled trials (RCTs), and two case reports on the subject of FMT as a treatment for depression [1, 4, 5, 6, 7, 8, 9, 10]. Further discussion of each study is presented below.

## 4 Pre‐Clinical Evidence

In the “mini review” of pre‐clinical studies of gut microbiota and FMT to treat depression, one study reported involved germ‐free rats colonized with fecal matter from depressed human donors who developed depressive behaviors, suggesting a causal role for gut dysbiosis [1, 4]. These rats displayed greater immobility and reduced struggling during the forced swim test (*p* = 0.013). The rats receiving FMT from depressed patients also showed increased abundance of Lachnospira and Ruminococcaceae and a reduction in Coprococcus, compared to rats receiving FMT from healthy donors [4]. The authors concluded that FMT can be used to alter specific gut bacteria which may influence mood regulation through the gut–brain axis.

In another study from the same review, FMT from chronically stressed rats induced neuroinflammatory changes and serotonin dysregulation in antibiotic treated healthy mice recipients [1, 5]. These mice likewise showed increased immobility in the tail‐suspension and forced‐swim tests and reduced hippocampal neurogenesis. The transfer also disrupted tryptophan–serotonin metabolism and reduced fluoxetine efficacy [5]. Another study in the review reported how transfer of healthy microbiota improved stress induced depressive behaviors in rats [1, 6]. Following FMT, there was an increase in brain derived serotonin levels and a reduction in IL‐1β and TNF‐α within the prefrontal cortex and hippocampus. This antidepressant effect was associated with reduced glial activation and suppression of the NLRP3 inflammasome pathway [6].

The authors of the review concluded that the findings illustrate that both depressive and antidepressant phenotypes can be modulated via FMT, reinforcing the mechanistic plausibility of gut‐targeted interventions [1].

## 5 Clinical Trials

Two randomized controlled trials and two case studies have been identified that considered the use of FMT to treat depression [7, 8, 9, 10].

The randomized controlled trial of FMT in depression was a pilot study of fifteen patients with moderate to severe MDD (as defined by DSM‐5 criteria), who were randomized to receive either FMT or placebo enemas in a 2:1 ratio over four consecutive days, followed by 26 weeks of follow‐up [7]. The primary outcome of the study was safety, and the study confirmed FMT was “feasible, acceptable, and well‐tolerated”. In addition, exploratory outcomes in the RCT revealed “a trend towards improved quality of life and gastrointestinal symptoms in the FMT group”, with depression scores measured via the Montgomery‐Åsberg Depression Rating Scale (MADRS) showing a non‐significant improvement (*p* = 0.67). However, limitations of this study were the small sample size and that depression measures were a secondary exploratory outcome rather than a primary study aim.

A second RCT randomized 18 patients with irritable bowel syndrome: diarrhea subtype (IBS‐D) to receive FMT or placebo capsules in a 1:1 ratio every other day for 3 days [8]. Depression and anxiety scores, measured using Hamilton Depression and Anxiety rating scales respectively, decreased over the 3‐month period after treatment (for depression *p* < 0.05 at 1 month, *p* < 0.01 at 2 months and *p* < 0.05 at 3 months; for anxiety *p* < 0.01 at 1,2 and 3 months post‐treatment). However, this study did not only have a small sample size, but it primarily considered a specific group of patients (those with IBS‐D), so findings regarding causality cannot be extrapolated to patients with depression alone.

One case report involved a 79‐year‐old woman with severe, treatment‐resistant depression receiving FMT [9]. Prior to FMT, the patient had received six months treatment with flupentixol, escitalopram, and melitracen, with no improvement in her depressive symptoms. Following a single FMT via gastroscopy (the sample donated by her 6‐year‐old grandson), the patient showed rapid and sustained remission of her depressive symptoms, with her Patient Health Questionnaire (PHQ‐9) score decreasing from 21 to 4 by 24 weeks and remaining stable at 12‐month follow‐up. This case report also noted a marked increase in Lachnospiraceae species, one of the SCFA‐producing families linked to anti‐inflammatory effects.

The second case reported use of FMT as adjunctive therapy in two patients with chronic depression [10]. Both patients were women, aged 53 and 58 years, with Hamilton Depression Rating Scale scores of 21 and 31 at baseline. Both patients received FMT as an adjunct to their “treatment as usual” (TAU): for Patient 1, this entailed lamotrigine, trazadone, bupropion, and vortioxetine; for Patient 2, TAU entailed lamotrigine, trazadone, escitalopram, lorazepam, pregabalin, chlorprothixene, and clotiapine. Both patients reported constipation at baseline, with Patient 1 receiving macrogol and psyllium husk in addition to TAU. After administration of FMT via oral capsules over a single 90‐min timeframe, both patients were followed up on a weekly basis, with post‐intervention measurements taken at 4 and 8 weeks. For Patient 1, symptoms of depression improved, indicated by a decreased HAMD‐score from 21 points at baseline to 9 points 4 weeks post‐intervention, but at the 8‐week follow‐up, the HAMD‐score increased to 19. For Patient 2, the HAMD‐score decreased from 31 to 10 points after 4 weeks and increased by two points after 8 weeks. The limitations of this case report were noted, including the short duration of follow‐up and impractical dosing (30 capsules to be consumed in one sitting).

## 6 Discussion

FMT has been proposed as a novel method to treat depression. Pre‐clinical research shows that microbiota transfer affects behavior, inflammation, and neurotransmitter levels. Clinical evidence remains limited, but the pilot trial reported is a critical starting point, demonstrating safety and feasibility while highlighting logistical issues such as delivery modality. Case reports offer additional evidence of efficacy, though these findings lack generalizability.

Although the literature search for this report was performed using a large and well‐renowned biomedical academic database, the inclusion criteria for the search (from 2016 only and in English language) as well as the consideration of only single database use may have limited the yield of results.

In addition, methodological variability across all trials such as differences in dosing, delivery, and donor screening limits comparability. Individual differences in baseline microbial profile limit reproducibility and may be responsible for the weaker effects observed in clinical trials compared to pre‐clinical models.

Multiple challenges hinder the clinical implementation of FMT. Standardization of preparation and delivery remains unresolved. Furthermore, FMT's “one‐size‐fits‐all” nature may overlook individualized microbiota needs.

FMT may hold therapeutic potential for major depressive disorder, but clinical evidence remains preliminary. While pre‐clinical studies strongly support its mechanistic plausibility, human trials are in early stages. Future large‐scale, placebo‐controlled RCTs focusing exclusively on depression are needed, ideally comparing FMT with existing antidepressants. Until then, FMT remains experimental but offers hope for patients with treatment‐resistant depression.

## Funding

The authors have nothing to report.

## Conflicts of Interest

The authors declare no conflicts of interest.

## Data Availability

Data sharing not applicable to this article as no datasets were generated or analysed during the current study.
